# Description of pain associated with persistent postoperative pain after total knee arthroplasty

**DOI:** 10.1038/s41598-024-66122-w

**Published:** 2024-07-02

**Authors:** Masayuki Koga, Akihisa Maeda, Shu Morioka

**Affiliations:** 1 Department of Neurorehabilitation, Graduate School of Health Sciences, Kio University, 4-2-2, Umaminaka, Koryo-cho, Kitakatsuragi-gun, Nara, 635-0832 Japan; 2 Department of Rehabilitation, Kawanishi City Medical Center, Hyogo, 666-0017 Japan; 3 Department of Rehabilitation, Kyowakai Hospital, Osaka, 564-0001 Japan; 4 Neurorehabilitation Research Center, Kio University, Nara, 635-0832 Japan

**Keywords:** Total knee arthroplasty, Description of pain, Persistent postoperative pain, Epidemiology, Chronic pain

## Abstract

After total knee arthroplasty (TKA), approximately 20% of patients experience persistent postoperative pain (PPP). Although preoperative and postoperative pain intensity is a relevant factor, more detailed description of pain is needed to determine specific intervention strategies for clinical conditions. This study aimed to clarify the associations between preoperative and postoperative descriptions of pain and PPP. Fifty-two TKA patients were evaluated for pain intensity and description of pain preoperatively and 2 weeks postoperatively, and the intensities were compared. In addition, the relationship between pain intensity and PPP at 3 and 6 months after surgery was analyzed using a Bayesian approach. Descriptions of arthritis (“Throbbing” and “aching”) improved from preoperative to 2 weeks postoperative. Several preoperative (“Shooting”, “Aching”, “Caused by touch”, “Numbness”) and postoperative (“Cramping pain”) descriptors were associated with pain intensity at 3 months postoperatively, but only “cramping pain” at 2 weeks postoperatively was associated with the presence of PPP at 3 and 6 months postoperatively. In conclusion, it is important to carefully listen to the patient’s complaints and determine the appropriate intervention strategy for the clinical condition during perioperative pain management.

## Introduction

Total knee arthroplasty (TKA) is an effective pain relief, cost-effective, and quality of life intervention for advanced-stage knee osteoarthritis (KOA)^1,2^. Postoperatively, it has also been reported to improve peripheral and central sensitization^3^. However, approximately 20% of patients experience persistent postoperative pain (PPP) lasting more than 3 months after TKA^4,5^. Pain intensity, in particular preoperative pain intensity, is relevant in these cases^6–8^. In addition, acute postoperative pain has also been reported as an associated factor^9–11^. Therefore, perioperative pain management is important to prevent PPP.

Pain involves multidimensional mechanisms and requires an individualized approach according to the factors involved^12^. In clinical situations, patients undergoing TKA express various descriptions of pain according to individual factors. For example, “throbbing” and “sharpness” are common complaints during the joint inflammation and postoperative inflammatory phases. Patients with nerve damage often express descriptions derived from sensory abnormalities such as “tingling,” “pricking,” and “numbness.” Thus, the description of pain contains information that reflects its etiology and is important for deciding the intervention strategy. Several studies have shown that there is a pathological classification by the description of pain^13–15^, and that the intervention effect differs according to its characteristics^14,16–18^. Although there have been reports analyzing the description of pain in the acute phase of TKA^19^, to the best of our knowledge, no studies have reported its relationship with PPP. Clarification of the changes before and after TKA and the description of pain associated with prolonged pain may assist in determining interventions that are appropriate for each patient’s condition during the perioperative period.

Therefore, in this study, we aimed to clarify changes in the description of pain before and after TKA and their association with PPP.

## Results

**Figure 1:**
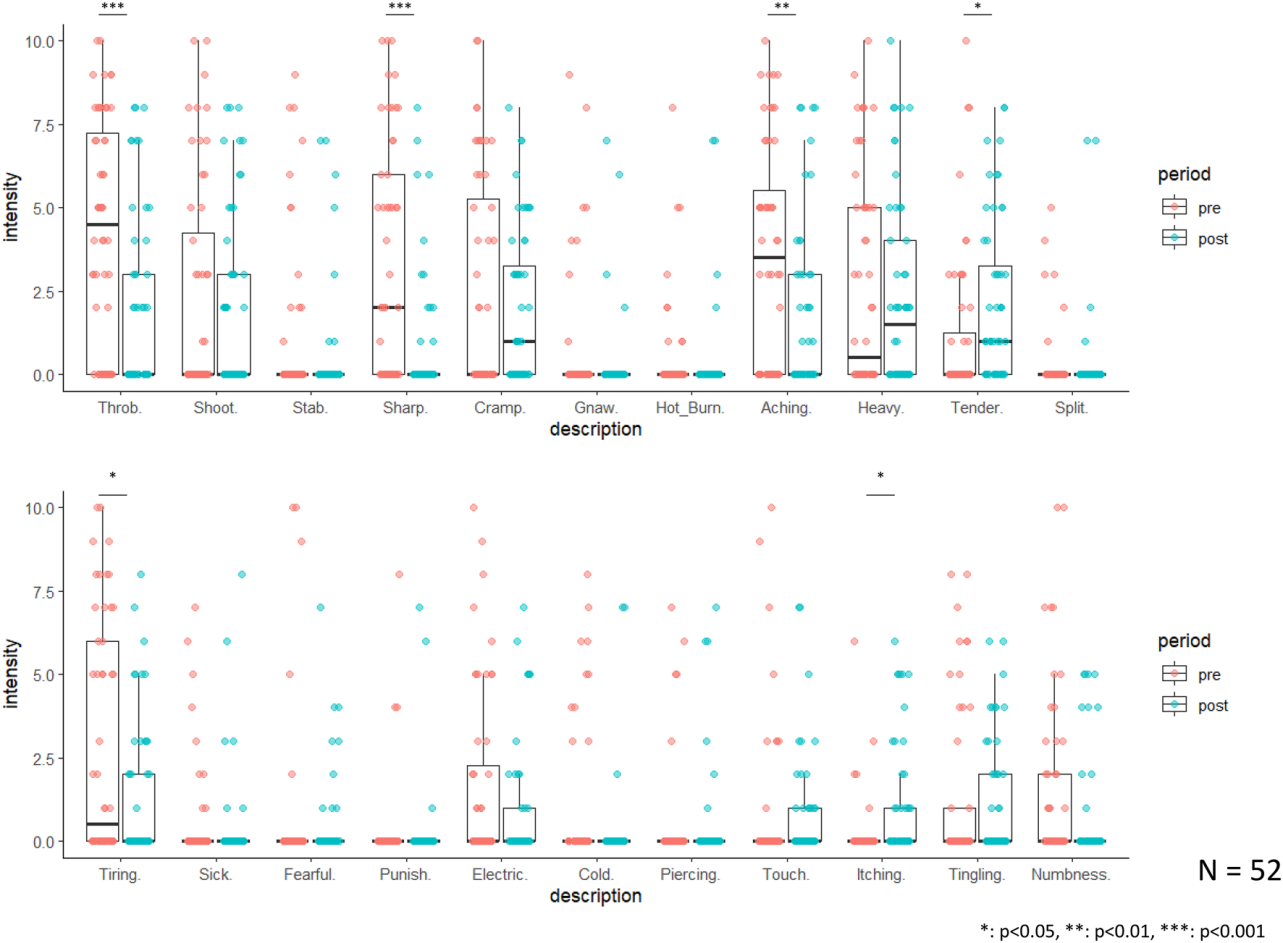
Description of pain preoperatively (red dot plot and left side of the boxplot) and 2 weeks postoperatively (green dot plot and right side of the boxplot).

Patient characteristics and preoperative/postoperative assessments are shown in Tables 1 and 2 and Figure 1. The Mann–Whitney U test and Chi–squared test / Fisher's Exact Test showed the pain duration (*p*=0.036), blood loss (*p*=0.002) and TKA type (*P*=0.045) significant differences in the presence or absence of PPP at 6 months postoperatively. The Friedman test and multiple comparisons (Bonferroni correction) test showed that the Numerical Rating Scale (NRS) score improved significantly from preoperative to 6 months postoperatively (*p*<0.001). The Wilcoxon signed-rank test showed significant postoperative improvement in the short-form McGill pain questionnaire-2 (SFMPQ-2) total score (*p* < 0.001) and the three subclasses (continuous pain, *p*=0.013; intermittent pain, *p* = 0.004; and affective pain, *p* = 0.013). Regarding the description of pain, four items (throbbing pain, *p* <0.001; sharp pain, *p*<0.001; aching pain, *p*=0.001; and tiring/exhausting, *p*=0.002) significantly improved, and two items (tender, *p*=0.025 and itching, *p*=0.013) significantly worsened.

**Table 1:** Characteristics of the participants.

Variables	All N = 52 (75 knees)	At 3 months			At 6 months		
		PPP N = 26 (38 knees)	Non-PPP N = 25 (35 knees)	*P* value^†^	PPP N = 9 (14 knees)	Non-PPP N = 42 (60 knees)	*P* value^†^
Age, years	76.2±8.3	75.5±8.7	76.7±8.4	0.678	74.8±6.3	76.6±8.8	0.347
Sex (Female/Male)	50/2	26/0	23/2	0.235	40/2	9/0	1.000
BMI, kg/m^2^	26.6±5.1	26.6±4.8	26.6±5.4	0.540	27.3±4.9	26.5±5.2	0.348
Pain duration, months	93.2±96.1	128.3±115.9	71.2±47.2	0.175	163.1±123.7	85.4±78.2	0.036
Operative side (Uni/Bil)	29/23	14/12	15/10	0.657	4/5	24/18	0.713
KL grade (III/IV)	19/56	7/31	11/24	0.197	4/10	15/45	0.745
Operative time, min	70.2±16.2	72.1±14.9	67.9±17.6	0.271	74.0±18.8	69.1±15.5	0.430
Blood loss, ml	17.1±18.4	16.9±15.9	17.3±21.5	0.484	18.3±3.2	16.8±20.2	0.002
Wound size, cm	13.3±1.5	13.5±1.4	13.1±1.7	0.134	13.1±1.4	13.3±1.5	0.729
TKA type (PS/CR/others)	51/20/4	26/10/2	23/10/2	1.000	6/6/2	44/14/2	0.045

Mean±standard deviation

^†^ assessed by Mann–Whitney U test or Chi-squared test or Fisher's Exact Test.

PPP, persistent postoperative pain; BMI, body mass index; Uni, unilateral; Bil, bilateral; KL grade; Kellgren-Lawrence grade.

**Table 2:** Comparison of the preoperative/postoperative description of pain.

Variables, median (IQR)	Pre	Post 2W	Post 3M	Post 6M	*P* value^†^
NRS	7 (5.75-9)^a^	4 (3-6)^b^	3 (1-3)^c^	1 (0-2)^d^	<0.001
Variables, median (IQR)	Pre		Post 2w		P value^‡^
SF-MPQ-2					
Total	37.5 (23.5-59)		14 (6.75-27.75)		<0.001
Continuous pain	13 (7-19.25)		8 (3-14.25)		0.013
Intermittent pain	6.5 (0-14.25)		2 (0-6)		0.004
Neuropathic pain	3 (0-7.25)		2.5 (0-5)		0.266
Affective pain	1 (0-7)		0 (0-3)		0.013
Description of pain					
Throbbing pain	4.5 (0-7.25)		0 (0-3)		<0.001
Shooting pain	0 (0-4.25)		0 (0-3)		0.813
Stabbing pain	0 (0-0)		0 (0-0)		0.177
Sharp pain	2 (0-6)		0 (0-0)		<0.001
Cramping pain	0 (0-5.25)		1 (0-3.25)		0.424
Gnawing pain	0 (0-0)		0 (0-0)		0.284
Hot-burning pain	0 (0-0)		0 (0-0)		0.479
Aching pain	3.5 (0-5.5)		0 (0-3)		0.001
Heavy pain	0.5 (0-5)		1.5 (0-4)		0.395
Tender	0 (0-3.25)		1 (0-3.25)		0.025
Splitting pain	0 (0-0)		0 (0-0)		0.766
Tiring/exhausting	0.5 (0-6)		0 (0-2)		0.002
Sickening	0 (0-0)		0 (0-0)		0.420
Fearful	0 (0-0)		(0-0)		0.645
Punishing/cruel	0 (0-0)		0 (0-0)		1.000
Electric-shock pain	0 (0-2.25)		0 (0-1)		0.114
Cold-freezing pain	0 (0-0)		0 (0-0)		0.123
Piercing	0 (0-0)		0 (0-0)		1.000
Pain caused by light touch	0 (0-0)		0 (0-1)		0.970
Itching	0 (0-0)		0 (0-1)		0.013
Tingling or pins and needles	0 (0-1)		0 (0-2)		0.799
Numbness	0 (0-2)		0 (0-0)		0.071

^†^assessed by Friedman test (post hoc Bonferroni correction), a>b>c>d.

^‡^assessed by Wilcoxon signed-rank test (post hoc Bonferroni correction).

Pre, preoperative; Post 2W, 2 weeks postoperative; Post 3M, 3 months postoperative; Post 6M, 6 months postoperative; IQR, interquartile range; NRS, Numerical Rating Scale; SFMPQ-2, short-form McGill Pain Questionnaire 2.

A Generalized Linear Mixed Model (GLMM) of Poisson distribution with NRS at 3 months postoperatively as the objective variable, individual differences (individual ID) as the random effect, and Bayesian estimation using the Markov chain Monte Carlo (MCMC) method (two models: SFMPQ-2 subclass and description of pain) were analyzed. The results showed that, in the description of the pain model, four preoperative items, shooting pain (estimate: 0.42, 95% confidence interval [CI] 0.06–0.80), aching pain (estimate: 0.50, 95% CI 0.02–1.04), caused by touch (estimate: −0.68, 95% CI −1.41–−0.01), and numbness (estimate: 0.42, 95% CI 0.05–0.78) [individual ID for random effects (estimate: 0.37, 95% CI 0.02–0.92)], and one postoperative item, cramping pain (estimate: 0.50, 95% CI 0.03–1.02) [individual ID for random effects (estimate: 0.44, 95% CI 0.03–0.96)] were significantly associated with the NRS at 3 months postoperatively (Table 3). GLMM with the NRS score at 6 months postoperatively as the objective variable did not yield convergence of the MCMC.

**Table 3:** GLMM for the association between NRS at 3 months and descriptions of pain.

Model	Variables	Preoperative		2 weeks postoperative	
		Estimate (95% CI)	Rhat	Estimate (95% CI)	Rhat
SF-MPQ-2 subclass	(Fixed effects)				
	Continuous pain	−0.17 (−0.53, 0.18)	1.00	0.25 (−0.18, 0.68)	1.00
	Intermittent pain	0.05 (−0.26, 0.35)	1.00	−0.27 (−0.82, 0.25)	1.00
	Neuropathic pain	0.21 (−0.07, 0.49)	1.00	0.28 (−0.16, 0.72)	1.00
	Affective pain	0.14 (−0.12, 0.38)	1.00	−0.12 (−0.59, 0.35)	1.00
	(Random effects)				
	Individual ID	0.35 (0.03,0.69)*	1.00	0.40 (0.05, 0.74)*	1.00
Pain Description	(Fixed effects)				
	Throbbing pain	−0.42 (−0.95, 0.04)	1.00	−0.19(−0.77, 0.34)	1.00
	Shooting pain	0.42 (0.06, 0.81)*	1.00	0.14 (−0.42, 0.68)	1.00
	Stabbing pain	−0.19 (−0.61, 0.19)	1.00	−0.38 (−1.48, 0.68)	1.00
	Sharp pain	0.38 (−0.03, 0.80)	1.00	0.33 (−0.65, 1.31)	1.00
	Cramping pain	−0.39 (−0.85, 0.03)	1.00	0.50 (0.03, 1.02)*	1.00
	Gnawing pain	−0.07 (−0.47, 0.32)	1.00	0.29 (−0.90, 1.52)	1.00
	Hot-burning pain	0.08 (−0.29, 0.44)	1.00	0.04 (−1.70, 1.74)	1.00
	Aching pain	0.50 (0.02, 1.04)*	1.00	−0.16 (−0.65, 0.33)	1.00
	Heavy pain	−0.15 (−0.63, 0.33)	1.00	0.04 (−0.40, 0.49)	1.00
	Tender	0.39 (−0.14, 0.94)	1.00	0.08 (−0.34, 0.50)	1.00
	Splitting pain	0.15 (−0.80, 1.08)	1.00	0.90 (−2.51, 4.58)	1.00
	Tiring/exhausting	0.06 (−0.34, 0.47)	1.00	0.10 (−0.40, 0.58)	1.00
	Sickening	−0.23 (−0.92, 0.40)	1.00	0.19 (−0.86, 1.24)	1.00
	Fearful	0.75 (−0.17, 1.79)	1.00	0.05 (−0.47, 0.52)	1.00
	Punishing/cruel	−0.38 (−1.80, 0.95)	1.00	−0.29 (−4.02, 3.20)	1.00
	Electric-shock pain	−0.33 (−1.80, 0.95)	1.00	0.13 (−0.52, 0.81)	1.00
	Cold-freezing pain	0.32 (−0.10, 0.76)	1.00	−0.37 (−2.01, 1.03)	1.00
	Piercing	−0.21 (−0.85, 0.42)	1.00	−1.05 (−2.49, 0.16)	1.00
	Pain caused by touch	−0.68 (−1.41, −0.01)*	1.00	0.01 (−0.78, 0.79)	1.00
	Itching	0.10 (−0.42, 0.61)	1.00	−0.10 (−0.47, 0.26)	1.00
	Tingling or pins and needles	−0.08 (−0.49, 0.31)	1.00	−0.29 (−0.75, 0.11)	1.00
	Numbness	0.42 (0.05, 0.78)*	1.00	0.27 (−0.00, 0.57)	1.00
	(Random effect)				
	Individual ID	0.37 (0.02, 0.92)*	1.00	0.44 (0.03, 0.96)*	1.00

^*^When the 95% CI did not cross zero, it was considered significant.

GLMM, Generalized Linear Mixture Model; NRS, Numerical Rating Scale; CI, confidence interval; SFMPQ-2, short-form McGill Pain Questionnaire 2; Individual ID, individual differences.

The association between the preoperative/postoperative 2-week description of pain and the presence or absence of persistent pain at 3 and 6 months postoperatively was analyzed with a Bernoulli distribution. As the GLMM did not converge with the MCMC, the Generalized Linear Model (GLM) was used to exclude random effects (Table 4). As a result, only cramping pain at 2 weeks postoperatively was associated with the presence of persistent pain at 3 months (estimate: 1.42, 95% CI 0.60–2.37) and 6 months (estimate: 0.95, 95% CI 0.21–1.78) postoperatively. None of the preoperative descriptions showed any significant associations.

**Table 4:** GLM for the association between persistent postoperative pain and descriptions of pain.

Model	Variables	PPP at 3 months		PPP at 6 months	
		Estimate (95% CI)	Rhat	Estimate (95% CI)	Rhat
Preoperative description	Shooting pain	0.31 (−0.36, 1.04)	1.00	−0.42 (−1.55, 0.49)	1.00
	Aching pain	0.42 (−0.19, 1.05)	1.00	0.56 (−0.24, 1.39)	1.00
	Pain caused by touch	−0.44 (−1.26, 0.36)	1.00	0.19 (−0.89, 1.22)	1.00
	Numbness	0.31 (−0.40, 1.05)	1.00	−0.18 (−1.21, 0.69)	1.00
Postoperative description	Cramping pain	1.42 (0.60, 2.37)*	1.00	0.95 (0.21, 1.78)*	1.00

^*^When the 95% CI did not cross zero, it was considered significant.

GLM, Generalized Linear Model; PPP, persistent postoperative pain; CI, confidence interval.

## Discussion

In this study, we focused on the description of pain, investigated changes before and after TKA, and examined their relationship with persistent pain at 3 and 6 months postoperatively. The main findings showed the following: (1) descriptions of pain, such as “throbbing,” “sharp,” “aching,” and “tiring/exhausting,” improved with TKA, while “tender” and “itching” worsened slightly; (2) some preoperative descriptions (“shooting,” “aching,” “caused by touch,” and “numbness”) and one postoperative description (“cramping pain”) were associated with NRS at 3 months; and (3) only postoperative “cramping pain” was associated with the presence of persistent pain at 3 and 6 months.

The median preoperative descriptions “throbbing” and “aching” were moderate to severe^20^, but improved significantly postoperatively. “Throbbing” and “aching” pains are caused by joint arthritis, suggesting that the joint problem has been improved by TKA^21–23^. Since pain is also associated with fatigue^24^, it is considered that those with “tiring/exhausting” pain descriptions showed improvements in pain intensity. On the other hand, those with pain descriptions of “tender” and “itching” showed slight worsening. Two weeks after surgery, when the wound healed and the staples was removed, the residual peripheral/central sensitization caused by the surgical invasion may have caused these descriptors. However, Graven-Nielsen et al. showed that peripheral/central sensitization improves approximately 5–28 weeks postoperatively^3^, and these temporarily worsening pain symptoms improve over time. In the SFMPQ-2 subscale, only neuropathic pain alone showed no improvement. As arthroplasty does not improve nerve problems, patients with preoperative neuropathic pain should be considered for perioperative treatment, which includes pharmacotherapy^18^ and electrical stimulation^25,26^.

The GLMM analysis of Poisson distribution (description of pain model) with individual ID as a random effect and 22 descriptions as fixed effects showed that several preoperative (“shooting pain,” “aching pain,” “caused by touch,” and “numbness”) and postoperative (“cramping pain”) descriptions were associated with the NRS at 3 months. However, the GLM analysis of the Bernoulli distribution showed that only postoperative “cramping pain” was associated with the presence of PPP at 3 and 6 months postoperatively.

The pain expression “cramping pain” is described when the muscles are over-contracted. This type of pain is typically caused by fatigue associated with overuse^27^. Muscle weakness after TKA has been attributed not only to peripheral skeletal muscles, but also to central nervous system control, including the spinal cord and cerebral cortex^28,29^. This may also contribute to the occurrence of cramping pain. Pain expressions reflecting motor control problems have also been observed in patients with phantom limb pain^17^, intractable pain after nerve injury^16^, and central post-stroke pain^30^. Some studies have reported that these patients show improvements with interventions for the sensory-motor system using mirror therapy and virtual reality environments^16,17,30^. In addition, interventions that consider the central nervous system for perioperative TKA/KOA, such as repetitive transcranial magnetic stimulation^31^, transcranial direct current stimulation^32^, neuromuscular electrical stimulations^33^, motor imagery^34^, have been reported to be effective. Therefore, it is necessary to verify whether such specific interventions that take into account central nervous system control can improve cramping pain and prevent persistent pain in the management after TKA.

This study had several limitations. First, psychological factors^6–8,35^, central sensitization^36^, and neuropathic pain^7^ associated with PPP were not evaluated in detail. In addition, characteristics of the participants, pain duration, blood loss, TKA type associate with absent or present PPP at 6 months postoperatively in this study. These factors are probably reflected in the individual ID that was found to be associated with pain intensity at 3 months postoperatively in the GLMM. Second, the description of pain at 3 and 6 months postoperatively was not measured. It remains to be clarified whether the same description of pain as at 2 weeks postoperatively was continued to be used. Future studies could analyze these associations by assessing catastrophic thinking^37–39^, anxiety and depression^40^, central sensitization-related symptoms^36,39,41,42^, and neuropathic pain^7^, as well as by examining pain descriptions at 3 and 6 months. Third, central/peripheral neuromuscular activity measurements, such as functional magnetic resonance imaging, motor-evoked potentials, and electromyography, were not performed. Future studies should include these techniques while considering the underlying neuromuscular activity.

This study focused on the description of pain and clarifying its association with PPP after TKA. We conclude that, in perioperative pain management, it is important to carefully listen to the patient's complaints and determine the appropriate intervention strategy for the clinical condition.

## Methods

### Participants

The participants were hospitalized patients who underwent primary unilateral or bilateral TKA at Kyowakai Hospital between April 2018 and December 2023. Participants received standardized anesthesia, surgery, postoperative pain management, and guideline-based physical therapy^43,44^. The exclusion criteria were dementia, higher brain dysfunction, inability to respond to the questionnaire adequately, and revision TKA. The study was approved by the Kyowakai Hospital Ethical Review Committee (approval number: Kyorin18-1), and all participants provided written informed consent in accordance with the Declaration of Helsinki.

### Measures

Participant characteristics (age, sex, body mass index [BMI], preoperative pain duration, unilateral or bilateral surgery, Kellgren-Lawrence [KL] grade, operative time, blood loss, wound size, and TKA type) were collected from patient charts. A few days before and two weeks after surgery when the wound is healed and the staples are removed, the NRS was used to assess pain intensity, and the SFMPQ-2 was used to description of pain. In addition, the NRS was obtained 3 and 6 months postoperatively via mail or telephone. The preoperative and 2-weeks postoperative NRS and SFMPQ-2 were assessed in all participant, but lack of contact resulted in one missing NRS score at postoperative 3 or 6 months.

The NRS is the most commonly used measure of pain intensity with an 11-point scale ranging from 0 (no pain) to 10 (worst imaginable pain). The minimal clinically important difference is 22% for acute pain^45^ and 33% for chronic pain^46^. In this study, persistent pain was defined as moderate or severe pain (NRS≥3) persisting at 3 or 6 months postoperatively^20,47,48^.

The SFMPQ-2 is used to assess the intensity and description of pain. It consists of 22 sensory expressions of pain, with each item rated on an NRS scale of 0–10. The higher the intensity of pain, the higher the total score. The SFMPQ-2 has four subclasses (continuous pain, intermittent pain, affective pain, and neuropathic pain)^49^.

### Statistical analysis

Characteristics of participants were compared using the Mann–Whitney U test for continuous variables and Chi-square test or Fisher’s exact test was used for categorical variables.

The NRS assessed the preoperative and 2 weeks/3 months/6 months postoperative scores and compared them using the Friedman test (post hoc Bonferroni correction). In addition, each item was compared using the Wilcoxon signed-rank test preoperatively and 2 weeks postoperatively.

The association between SFMPQ-2 results preoperatively and 2 weeks postoperatively and pain intensity at 3 and 6 months postoperatively was analyzed by GLMM with individual ID as a random effect. A Bayesian estimation of the posterior distribution was performed using the MCMC algorithm^50^. MCMC is a method for obtaining an approximate solution to the integral through Monte Carlo integration using random numbers generated by a Markov chain, which enables a reasonable estimation for small sample sizes and missing values. The Z-score for each item was used in the analysis. An uninformative prior distribution was used, the number of iterations was set to 2000, burn-in period to 1000, number of chains to four, and 95% CI were given. Rhat values were used to evaluate MCMC convergence, wherein a Rhat value of <1.1 suggested good convergence.

We created two models in the analysis: (1) SFMPQ-subclass: continuous pain, intermittent pain, neuropathic pain, and affective pain; and (2) description of pain: “throbbing pain,” “shooting pain,” “stabbing pain,” “sharp pain,” “cramping pain,” “gnawing pain,” “hot-burning pain,” “aching pain,” “heavy pain,” “tender,” “splitting pain,” “tiring/exhausting,” “sickening,” “fearful,” “punishing/cruel,” “electric-shock pain,” “cold-freezing pain,” “piercing,” “pain caused by touch,” “itching,” “tingling or pins and needles,” and “numbness.” The association between each model and the NRS at 3 and 6 months postoperatively was analyzed using a Poisson distribution. Furthermore, the associated preoperative and postoperative items were analyzed by a Bernoulli distribution with the presence or absence of persistent pain (NRS≥3) with 3 and 6 months postoperatively as the objective variable. Statistical analyses were performed using the R ver. 4.1.3.

## Data Availability

The datasets used and/or analyzed during the current study available from the corresponding author on reasonable request.

## References

[1] Price AJ (2018). Knee replacement. Lancet.

[2] Dakin H (2012). Rationing of total knee replacement: a cost-effectiveness analysis on a large trial data set. BMJ Open.

[3] Graven-Nielsen T, Wodehouse T, Langford RM, Arendt-Nielsen L, Kidd BL (2012). Normalization of widespread hyperesthesia and facilitated spatial summation of deep-tissue pain in knee osteoarthritis patients after knee replacement. Arthritis Rheum..

[4] Wylde V (2018). Chronic pain after total knee arthroplasty. EFORT Open Rev..

[5] Beswick AD, Wylde V, Gooberman-Hill R, Blom A, Dieppe P (2012). What proportion of patients report long-term pain after total hip or knee replacement for osteoarthritis? A systematic review of prospective studies in unselected patients. BMJ Open.

[6] Olsen U (2023). Factors correlated with pain after total knee arthroplasty: A systematic review and meta-analysis. PLoS One.

[7] Rice DA (2018). Persistent postoperative pain after total knee arthroplasty: a prospective cohort study of potential risk factors. Br. J. Anaesth..

[8] Lewis GN, Rice DA, McNair PJ, Kluger M (2015). Predictors of persistent pain after total knee arthroplasty: a systematic review and meta-analysis. Br. J. Anaesth..

[9] Lavand’homme PM, Grosu I, France M-N, Thienpont E (2014). Pain trajectories identify patients at risk of persistent pain after knee arthroplasty: An observational study. Clin. Orthop. Relat. Res..

[10] Thomazeau J (2016). Predictive factors of chronic post-surgical pain at 6 months following knee replacement: Influence of postoperative pain trajectory and genetics. Pain Phys..

[11] Imai R (2021). Using a postoperative pain trajectory to predict pain at 1 year after total knee arthroplasty. Knee.

[12] Chimenti RL, Frey-Law LA, Sluka KA (2018). A mechanism-based approach to physical therapist management of pain. Phys. Ther..

[13] Freeman R, Baron R, Bouhassira D, Cabrera J, Emir B (2014). Sensory profiles of patients with neuropathic pain based on the neuropathic pain symptoms and signs. Pain.

[14] Uragami S (2024). Prognosis of pain after stroke during rehabilitation depends on the pain quality. Phys. Ther..

[15] Shigetoh H, Nishi Y, Osumi M, Morioka S (2022). The pain intensity/quality and pain site association with muscle activity and muscle activity distribution in patients with chronic low back pain: Using a generalized linear mixed model analysis. Pain Res. Manag..

[16] Sumitani M (2008). Mirror visual feedback alleviates deafferentation pain, depending on qualitative aspects of the pain: A preliminary report. Rheumatology.

[17] Osumi M (2019). Characteristics of phantom limb pain alleviated with virtual reality rehabilitation. Pain Med..

[18] Bouhassira D (2014). Neuropathic pain phenotyping as a predictor of treatment response in painful diabetic neuropathy: data from the randomized, double-blind COMBO-DN study. Pain.

[19] Wylde V, Rooker J, Halliday L, Blom A (2011). Acute postoperative pain at rest after hip and knee arthroplasty: Severity, sensory qualities and impact on sleep. Orthop. Traumatol. Surg. Res..

[20] Collins SL, Moore RA, McQuay HJ (1997). The visual analogue pain intensity scale: What is moderate pain in millimetres?. Pain.

[21] Wagstaff S, Smith OV, Wood PH (1985). Verbal pain descriptors used by patients with arthritis. Ann. Rheum. Dis..

[22] Englund M (2007). Effect of meniscal damage on the development of frequent knee pain, aching, or stiffness. Arthritis Rheum..

[23] Tanaka N, Sakahashi H, Sato E, Hirose K, Isima T (2003). Influence of the infrapatellar fat pad resection in a synovectomy during total knee arthroplasty in patients with rheumatoid arthritis. J. Arthroplasty.

[24] Yamada K (2022). The temporal relation between pain and fatigue in individuals receiving treatment for chronic musculoskeletal pain. BMC Musculoskelet. Disord..

[25] Gibson, W., Wand, B. M. & O’Connell, N. E. Transcutaneous electrical nerve stimulation (TENS) for neuropathic pain in adults. *Cochrane Database Syst. Rev.* 9, CD011976 (2017). 10.1002/14651858.CD011976.pub2 PMC6426434 28905362

[26] Nishi Y (2022). A novel form of transcutaneous electrical nerve stimulation for the reduction of dysesthesias caused by spinal nerve dysfunction: A case series. Front. Hum. Neurosci..

[27] Schwellnus MP (2009). Cause of exercise associated muscle cramps (EAMC)–altered neuromuscular control, dehydration or electrolyte depletion?. Br. J. Sports Med..

[28] Lepley AS, Lepley LK (2022). Mechanisms of arthrogenic muscle inhibition. J. Sport Rehabil..

[29] Paravlic AH, Kovač S, Pisot R, Marusic U (2020). Neurostructural correlates of strength decrease following total knee arthroplasty: A systematic review of the literature with meta-analysis. Bosn. J. Basic Med. Sci..

[30] Corbetta D, Sarasso E, Agosta F, Filippi M, Gatti R (2018). Mirror therapy for an adult with central post-stroke pain: A case report. Arch Physiother.

[31] Nguyen J-P (2019). The value of high-frequency repetitive transcranial magnetic stimulation of the motor cortex to treat central pain sensitization associated with knee osteoarthritis. Front. Neurosci..

[32] Borckardt JJ (2013). Transcranial direct current stimulation (tDCS) reduces postsurgical opioid consumption in total knee arthroplasty (TKA). Clin. J. Pain.

[33] Yoshida Y, Ikuno K, Shomoto K (2017). Comparison of the effect of sensory-level and conventional motor-level neuromuscular electrical stimulations on quadriceps strength after total knee arthroplasty: A prospective randomized single-blind trial. Arch. Phys. Med. Rehabil..

[34] Li R, Du J, Yang K, Wang X, Wang W (2022). Effectiveness of motor imagery for improving functional performance after total knee arthroplasty: A systematic review with meta-analysis. J. Orthop. Surg. Res..

[35] Khatib Y, Madan A, Naylor JM, Harris IA (2015). Do psychological factors predict poor outcome in patients undergoing TKA? A systematic review. Clin. Orthop. Relat. Res..

[36] Kim, M. S., Kim, J. J., Kang, K. H., Kim, M. J. & In, Y. Diagnosis of central sensitization and its effects on postoperative outcomes following total knee arthroplasty: A systematic review and meta-analysis. *Diagnostics* (Basel) 12, (2022). 10.3390/diagnostics12051248 PMC9141391 35626402

[37] Burns LC (2015). Pain catastrophizing as a risk factor for chronic pain after total knee arthroplasty: a systematic review. J. Pain Res..

[38] Hirakawa Y, Hara M, Fujiwara A, Hanada H, Morioka S (2014). The relationship among psychological factors, neglect-like symptoms and postoperative pain after total knee arthroplasty. Pain Res. Manag..

[39] Hasegawa M, Tone S, Naito Y, Sudo A (2022). Preoperative pain catastrophizing affects pain outcome after total knee arthroplasty. J. Orthop. Sci..

[40] Duivenvoorden T (2013). Anxiety and depressive symptoms before and after total hip and knee arthroplasty: A prospective multicentre study. Osteoarthritis Cartilage.

[41] Koga M, Shigetoh H, Tanaka Y, Morioka S (2022). Characteristics of clusters with contrasting relationships between central sensitization-related symptoms and pain. Sci. Rep..

[42] Shigetoh H, Koga M, Tanaka Y, Hirakawa Y, Morioka S (2024). Characterizing clinical progression in patients with musculoskeletal pain by pain severity and central sensitization-related symptoms. Sci. Rep..

[43] Jette DU (2020). Physical therapist management of total knee arthroplasty. Phys. Ther..

[44] Dávila Castrodad IM (2019). Rehabilitation protocols following total knee arthroplasty: A review of study designs and outcome measures. Ann Transl Med.

[45] Olsen MF (2017). Pain relief that matters to patients: Systematic review of empirical studies assessing the minimum clinically important difference in acute pain. BMC Med..

[46] Salaffi F, Stancati A, Silvestri CA, Ciapetti A, Grassi W (2004). Minimal clinically important changes in chronic musculoskeletal pain intensity measured on a numerical rating scale. Eur. J. Pain.

[47] Lewis GN (2020). A higher grey matter density in the amygdala and midbrain is associated with persistent pain following total knee arthroplasty. Pain Med..

[48] Edwards RR (2022). Multimodal prediction of pain and functional outcomes 6 months following total knee replacement: A prospective cohort study. BMC Musculoskelet. Disord..

[49] Dworkin RH (2009). Development and initial validation of an expanded and revised version of the Short-form McGill Pain Questionnaire (SF-MPQ-2). Pain.

[50] Cowles MK, Carlin BP (1996). Markov chain monte carlo convergence diagnostics: A comparative review. J. Am. Stat. Assoc..

